# Ultra-Broadband Absorption from 750.0 nm to 5351.6 nm in a Novel Grating Based on SiO_2_-Fe-Sandwich Substrate

**DOI:** 10.3390/ma12121892

**Published:** 2019-06-12

**Authors:** Zhaofeng Wu, Hanqing Liu, Hui Yang, Jibin Liu, Peiguo Liu

**Affiliations:** 1 College of Electronic Science and Technology, National University of Defense Technology, Changsha 410073, China; wuzhaofeng13@nudt.edu.cn (Z.W.); liujibin@nudt.edu.cn (J.L.); pg731@126.com (P.L.); 2 Bureau of Meteorology of Guangdong Province, Guangzhou 510530, China; yh128@163.com

**Keywords:** filling factor, grating, surface plasmon, ultra-broadband absorption

## Abstract

In this paper, we design a three-part-period grating based on alternating Fe/SiO${}_{2}$ sandwich structure, which can achieve an ultra-broadband absorption from 750.0 nm to 5351.6 nm. In particular, the absorbing efficiency can reach to more than 95% within 2158.8 nm, which is due to the well impedance matching of Fe with the free space, as well as due to the excitation of localized surface plasmon resonance and surface propagation plasmon resonance in the proposed structure. Furthermore, multiple period gratings are also discussed to broaden the absorption band. These results are very promising for applications in high-performance photovoltaics, nonlinear optics devices and protective equipment for laser weapons.

## 1. Introduction

As a fundamental optical process, absorption for electromagnetic wave plays a significantly important role in a wide variety of applications such as photovoltaics [1,2,3], surface enhanced Raman spectroscopy [4], plasmonic sensors [5,6,7], nonlinear optics and spectral filters [8,9]. In the past decade, it has been shown both theoretically and experimentally that absorbers based on plasmonic nanostructures and metamaterials can achieve spectrally selective absorption bands from the visible to the microwave band with a near-perfect efficiency resulting from the resonances, which also show merits such as low polarization and angle dependencies [10,11,12,13,14]. However, even if unity absorption can be realized at the resonance frequencies by using specific building blocks of certain geometries, actual structures still suffer from the limitation of absorbing bandwidth due to the narrow band resonance. A common solution for broadening the absorption band is via designing complex or multilayer structures that aim to overlap multiple resonances at the neighboring frequencies [15,16,17], and expect to result in complicated crafts and increased manufacturing costs. In this case, it is still a challenge to achieve an effective absorber with ultra-broadband absorption as well as simple manufacture.

In this letter, we have designed and numerically investigated a novel absorber with an ultra-broadband absorption from 750.0 nm to 5351.6 nm. It is realized by a three-part-period grating based on the SiO${}_{2}$-Fe-sandwich substrate (SFSS). Compared with the most commonly used metals such as Au, Fe can be much more beneficial for achieving the impedance matching with free space over a broad frequency band [18]. Meanwhile, the number of Fe layers, as well as thicknesses of Fe and SiO${}_{2}$ layers, are discussed to enlarge the multiple propagation surface plasmon (PSP) resonances in adjacent SiO${}_{2}$ layers and then improve the absorbing capacity in visible and near-infrared regions. Furthermore, the filling factors of three-part-period grating are optimized to enhance the localized surface plasmon (LSP) resonance and improve the absorption in mid-infrared, which is analytically demonstrated with the grating Fourier harmonics model. Thus, these properties clearly indicate our proposed structure as a perfect choice for ultra-broadband absorber in applications.

## 2. Design for Ordinary Grating Based on SFSS

**Figure 1:**
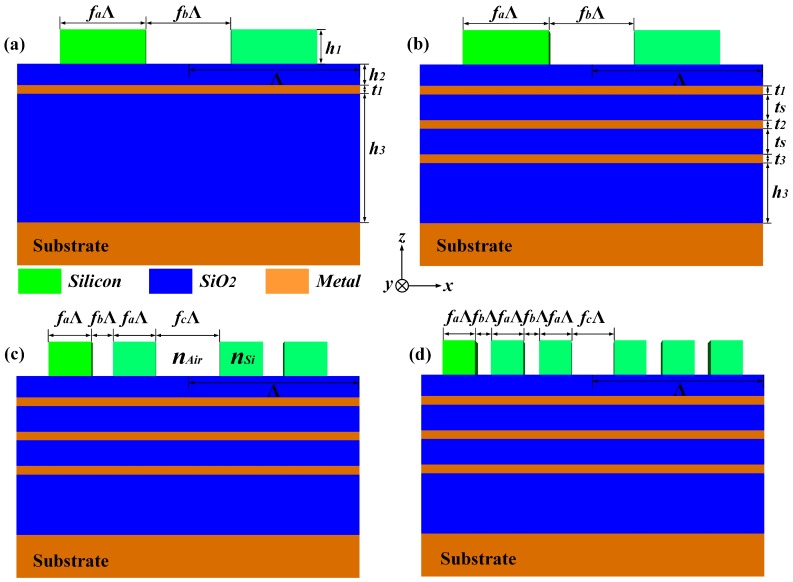
Schematic diagram of the ordinary silicon grating based on SiO${}_{2}$-metal-sandwich substrate consists of (**a**) single metal layer; (**b**) three Fe layers; schematic diagram of the proposed (**c**) two-part-period grating and (**d**) three-part-period grating based on SFSS.

**Figure 2:**
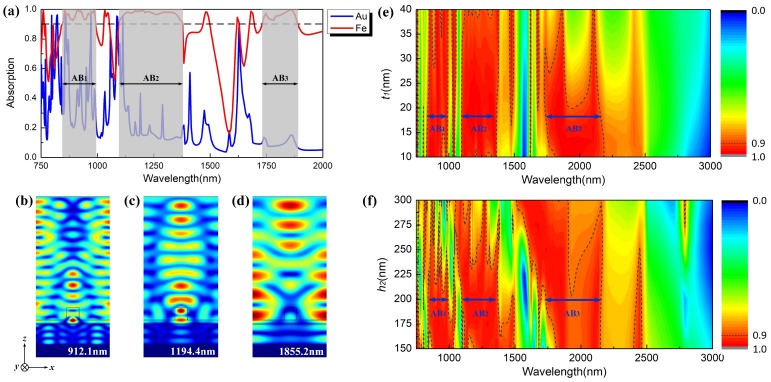
(**a**) absorption spectrum of the ordinary grating supported by SGSS and SFSS; Electric field of the proposed structure in the vertical direction at the wavelength of (**b**) 912.1 nm; (**c**) 1194.4 nm and (**d**) 1855.2 nm, respectively; absorption spectra of the single-layer GSFS varying with (**e**) thickness $t_{1}$ of Fe layer and (**f**) thickness $h_{2}$ of the upper SiO${}_{2}$ layer.

To begin with, as schematically shown in Figure 1a,b, we discuss the absorption performance of the ordinary grating placed on a thin SiO${}_{2}$ layer which is supported by the SiO${}_{2}$-metal-sandwich substrate. The period width Λ is chosen to ensure that the proposed structure is a sub-wavelength device for the operating wavelength. Define the filling factors of the silicon grating and free space gap as $f_{a}$ and $f_{b}$, respectively, and the geometrical height of grating as $h_{1}$. In Figure 1a, there is only one layer metal with thickness $t_{1}$ which exists in the multiple substrate. The thicknesses of upper SiO${}_{2}$ layer and bottom SiO${}_{2}$ layer are defined as $h_{2}$ and $h_{3}$, respectively. As the incident light illuminates on the structure, the optical response of metal surface can be easily understood by examining its dielectric function, which, as a good approximation, can be described by the Drude model [19]:

(1) $$\begin{array}{c} \varepsilon \left(\omega \right)=1+\frac{i\tau \omega_{p}^{2}}{\omega \left(1-i\omega \tau \right)}, \end{array}$$

where ω is the angular frequency of the radiation, $\omega_{p}$ is the plasma frequency, and τ is the relaxation time of the electrons. Below $\omega_{p}$, the real part of ε is negative and only evanescent waves are allowed in the metal. The penetration of light into the metal is characterized by the skin depth $\delta =c/2\omega \sqrt{\varepsilon }$, and the transmission through a flat metal layer of thickness $t_{1}$ can be expressed as $T=\exp\left(-t_{1}/\varepsilon \right)$. Thus, the value of $t_{1}$ needs to be appropriate for the aim of effectively absorbing the incident light, while the metal layer at the bottom needs to be thick enough to be regarded as a Bragg reflector. At the same time, the silicon grating can also provide the in-plane momentum required for the incident radiation (if appropriately polarized) to excite a surface plasmon polariton (SPP), resulting in strong optical absorption [20]. A normally incident Transverse Magnetic (TM) light is incident along the negative *y*-axis with the polarization along with the *x*-axis. Assume that the relevant geometrical dimensions in Figure 1a are Λ = 3600 nm, $f_{a}$ = 0.167, $h_{1}$ = 600 nm, $h_{2}$ = 200 nm, and $t_{1}$ = 1200 nm. Here, as shown in Figure 2a, through the simulation with finite element method on the commercial software COMSOL V5.3, the absorption spectrum of the proposed grating based on SiO${}_{2}$-Au-sandwich substrate (SASS) and SiO${}_{2}$-Fe-sandwich substrate (SFSS) are plotted over the wavelength range from 750 nm to 2000 nm, respectively. It can be obtained that, for SASS, the absorption peaks are sharp and discrete and only a tiny minority of them in the visible and near-infrared can surpass 90%. On the contrary, for SFSS, we can find that there are three main absorption bands that can satisfy the average absorption up to 95%, respectively located at 843.4 nm to 990.1 nm (AB${}_{1}$), 1095.9 nm to 1377.8 nm (AB${}_{2}$), and 1729.9 nm to 1884.5 nm (AB${}_{3}$).

To better explain the grating in the absorption performances using Fe, we also give a detailed calculation and analysis based on the impedance transformation method. Here, an effective medium theory is utilized to analyze the impedance matching condition [21]. The relation between the *S* parameters and impedance *Z* can be expressed as:

(2) $$\begin{array}{c} S_{21}=S_{12}=\frac{1}{\cos\left(nkd\right)-\frac{i}{2}\left(Z+\frac{1}{2}\right)\sin\left(nkd\right)}, \end{array}$$

(3) $$\begin{array}{c} S_{11}=S_{22}=\frac{i}{2}\left(\frac{1}{Z}-Z\right)\sin\left(nkd\right), \end{array}$$

where $S_{11}$, $S_{21}$, $S_{12}$, $S_{22}$, *n*, *k*, and *d* are *S* parameters, the effective refractive index, the wave vector, and thickness of the structure, respectively. The absorption rate can be regarded as:

(4) $$\begin{array}{c} A=1-T-R=1-{\left|S_{21}\right|}^{2}-{\left|S_{11}\right|}^{2}. \end{array}$$

In the proposed structure, we have $S_{12}$ = 0; as a result, to obtain a broadband absorption spectrum, the ideal condition is Z=1, $S_{11}=0$, and then A=1. According to previous research, the skin depth of Fe is about twice as large as Au, which means that the impedance of SFSS can satisfy the impedance matching condition over a wide wavelength range, and thus it is easy to observe a higher level of absorption due to a better impedance matching between the SFSS and the free space, as shown in Figure 2a.

Furthermore, to have a deeper insight into the wideband absorption mechanism, we also plot the electric field distributions of the SFSS at 912.1 nm, 1194.4 nm, and 1855.2 nm as shown in Figure 2b–d, respectively. In Figure 2b,c, the electric field focus on the upper SiO${}_{2}$ layer between silicon grating and Fe layer, which means that the absorption mainly originates from the excitation of Local Surface Plamons (LSP) resonance mode in the upper SiO${}_{2}$ layer. However, the electric field in Figure 2d distributes not only in the the upper SiO${}_{2}$ layer but also in the bottom SiO${}_{2}$ layer, thus in this case the absorption is mainly attributed by the hybridization between LSP mode and Polar Surface Plasmons (PSP) mode.

**Figure 3:**
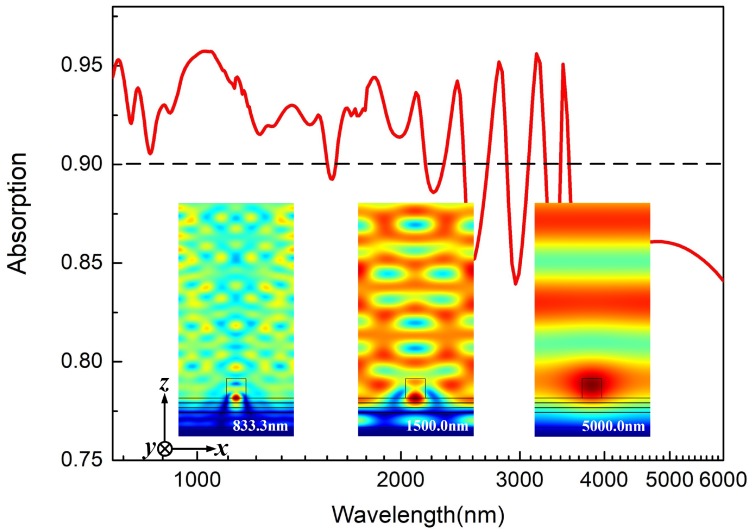
Absorption spectrum of ordinary grating in Figure 1b supported by three-layer SFSS over the wavelength range from 750 nm to 6000 nm; The illustrations are electric field distribution in the vertical direction at the wavelength of 833.3 nm, 1500.0 nm and 5000.0 nm, respectively.

In addition, we also discuss the influences of parameters $t_{1}$ and $h_{2}$ to the absorption performance of the ordinary grating supported by SFSS, respectively. As shown in Figure 2e, the bandwidths of AB${}_{1}$ and AB${}_{2}$ keep invariable as $t_{1}$ increases from 10 nm to 40 nm, while the bandwidth of AB${}_{3}$ breaks up into two narrow bands gradually when $t_{1}\geq 21$ nm. Since the variation of the thickness of Fe layer in SFSS can easily tune the PSP mode in the bottom SiO${}_{2}$ layer, it is verified that only AB${}_{3}$ is connected with the PSP mode. Meanwhile, all three of these bands split to several parts as $h_{2}$ increases from 150 nm to 300 nm as shown in Figure 2f, and, particularly, AB${}_{1}$ and AB${}_{2}$ are nearly disappearing when $h_{2}\geq 250$ nm. Hence, all three of these bands can be efficiently modulated by the LSP mode in the upper SiO${}_{2}$ layer. These relationships are well matched with the electric fields as plotted in Figure 2b–d. To further broaden the absorption band, we also calculate the absorption performance of the proposed structure with multiple Fe layers in SFSS. Since LSP mode excited by the incident light acts as the “bright” mode and PSP mode excited by the field confined in the multiple layers acts as the “dark” mode, the proposed structure can be interpreted as a series of harmonic oscillators driven by the external forces [22]. The absorption peaks mainly consist of bright and dark oscillators and the number of them can increase due to the resonance coupling between PSP of neighboring SiO${}_{2}$ layers. As shown in Figure 1b, the thicknesses of three Fe layers in the proposed structure are defined as $t_{1}=20$ nm, $t_{2}=20$ nm, and $t_{3}=30$ nm, respectively, and the thickness of SiO${}_{2}$ is defined as $t_{s}=250$ nm. Compared with Figure 2a, in Figure 2c, this multiple structure can nearly realize more than 90% absorption for incident light over the band from 750.0 nm to 2176.3 nm; meanwhile, in the longer wavelength, the absorption can still remain 85% up to 5538.1 nm. The illustrations in Figure 3 show the electric field distribution in the vertical direction at different wavelengths. At 5000.0 nm, when the parameters of the grating are properly designed, phase matching between the guided mode of the grating and the incident light is enabled and consequently the LSP mode of the grating is excited, which leads to significant improvement of the optical electric field both inside and around the strips of grating. On the other hand, at 1500.0 nm and 833.3 nm, the optical electric field mainly exists in the SFSS, especially at the interface between SiO${}_{2}$ and Fe layers. This should be attributed to the PSP mode which occurs when the reciprocal lattice vector of the grating is equal to the wave vector of the PSP mode of the multiple structure. Both the LSP and PSP modes are simultaneously modulated by the periodic modulation of refractive index in SFSS and the periodicity of dielectric grating. Hence, the optical electric field resonates to the incident light at both the parallel and perpendicular directions, which in turn improves the optical electric field both inside and around the multiple structure.

## 3. Design for Two-Part-Period and Three-Part-Period Gratings Based on SFSS

**Figure 4:**
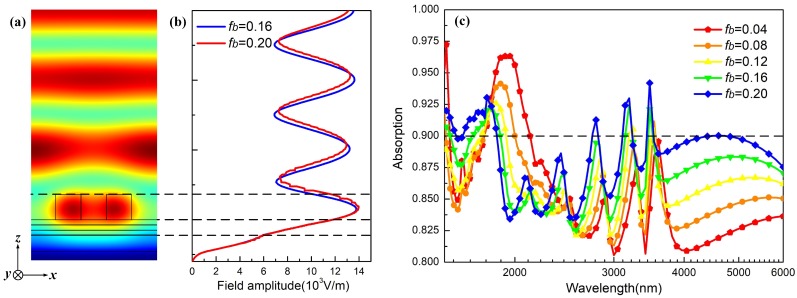
Electric field distribution (**a**) and amplitude of the electric modal fields (**b**) of the proposed two-part-period grating based on three-layer SFSS in the vertical direction at the wavelength of 2833.9 nm; (**c**) absorption spectrum over the range from 1500 nm to 6000 nm with different values of fill factor $f_{b}$.

According to the simulation of multiple SFSS above, in order to broaden the absorption band of our proposed structure to mid-infrared, the LSP resonance mode in the strips of grating need to be enhanced. Here, we resort to a different LSP configuration, in which each period is composed of two grating ridges with identical width, as shown in Figure 1c. In essence, this kind of grating enables a rich set of Fourier harmonics with concomitant emergence of additional spectral features not available for the classic period grating as shown in Figure 1a,b. The periodic constant, the thickness of Fe and SiO${}_{2}$ layers of the proposed two-part-period grating are equal to the corresponding values in Figure 1b. Assume that the total width of the two grating grooves in each period is kept constant, that is, $f_{b}+f_{c}=0.4$. According to the rigorous coupled-wave theory [22], the grating Fourier harmonics $\varepsilon_{n}$ control the amplitude of evanescent diffraction fields and are responsible for the mutual interaction of the evanescent diffraction fields, which have the following forms [23]:

(5) $$\begin{array}{l} \varepsilon_{0}=\left(1-f_{b}-f_{c}\right)n_{Si}^{2}+\left(f_{b}+f_{c}\right)n_{Air}^{2},\\ \varepsilon_{n}=\left(n_{Si}^{2}-n_{Air}^{2}\right)\frac{\sin\left[n\pi \left(1-f_{c}\right)\right]-\sin\left(n\pi f_{b}\right)}{n\pi }, \end{array}$$

where $n_{Si}$ and $n_{Air}$ represent the refractive indexes of silicon and air, respectively. *n* is equal to ±1,±2,…±N…. As a result, by modulating the value of the fill factor $f_{b}$ and $f_{c}$, we can achieve the variation of LSP mode in the two-part-period grating and then tune the bandwidth of absorption. Figure 4a displays the electric field at the resonate wavelength 2833.9 nm for the fill factor $f_{b}=$ 0.2. As expected, the enhanced electric field is mainly located in the strips of grating, which indicates that the absorption performance at the mid-infrared region originates from the excitation of LSP mode. As shown in Figure 4b, the amplitude of electric field cyclical distributes in free space, while in grating it is intensive to 1.4 × $10^{4}$ V/m and then reduces rapidly in the SSFS. Particularly, when the value varies from 0.2 to 0.16, it is observed that the amplitude of the electric modal field in grating decreases, but the amplitude of the electric modal field in SFSS remains invariable, which demonstrates that the LSP mode in grating can be modulated by changing fill factor $f_{b}$. Figure 4c shows the absorption spectrum with different fill factors, which can be divided into two parts: one is from 1500 nm to 2500 nm and the other is from 2500 nm to 6000 nm. In the first part, the absorption is ascribed to the hybridization between the LSP resonance in the strip of grating and the PSP resonances in the SiO${}_{2}$ layer, thus the increase of $f_{b}$ makes the variation of absorption amplitude fluctuate smoothly. Note that this does not mean that the absorption efficiency in this system reduces as $f_{b}$ goes up because the positive relationship between the amplitude of electric field and $f_{b}$ as shown in Figure 4b is only appropriate for mid-infrared. Furthermore, it can be obtained that the absorption to incident light can reach 98.7% at about 1692.3 nm when $f_{b}$ is equal to 0.04. On the other hand, in the second wavelength part, the relationship between absorption amplitude and fill factor becomes more distinct since the resonance only relies on the LSP mode. In this case, the absorption can reach 94.2% at about 3473.7 nm when $f_{b}$ is 0.20. Compared with the origin structure in Figure 1b, we can only obtain absorption from 750.0 nm to 1850.5 nm in this two-part-period grating based on SFSS by modulating the fill factor, thus these results still do not satisfy our aim to realize an ultra-broadband absorption.

Furthermore, on the basis of the proposed structures above, we design another three-part-period grating with SFSS as shown in Figure 1d. The grating Fourier harmonics $\varepsilon_{n}^{*}$ of this kind of structure can be expressed as:

(6) $$\begin{array}{l} \varepsilon_{0}^{*}=\left(1-2f_{b}-f_{c}\right)n_{Si}^{2}+\left(2f_{b}+f_{c}\right)n_{Air}^{2},\\ \varepsilon_{n}^{*}=\left(n_{Si}^{2}-n_{Air}^{2}\right)\frac{\sin\left[n\pi \left(1-f_{c}\right)\right]-\sin\left(2n\pi f_{b}\right)}{n\pi }. \end{array}$$

**Figure 5:**
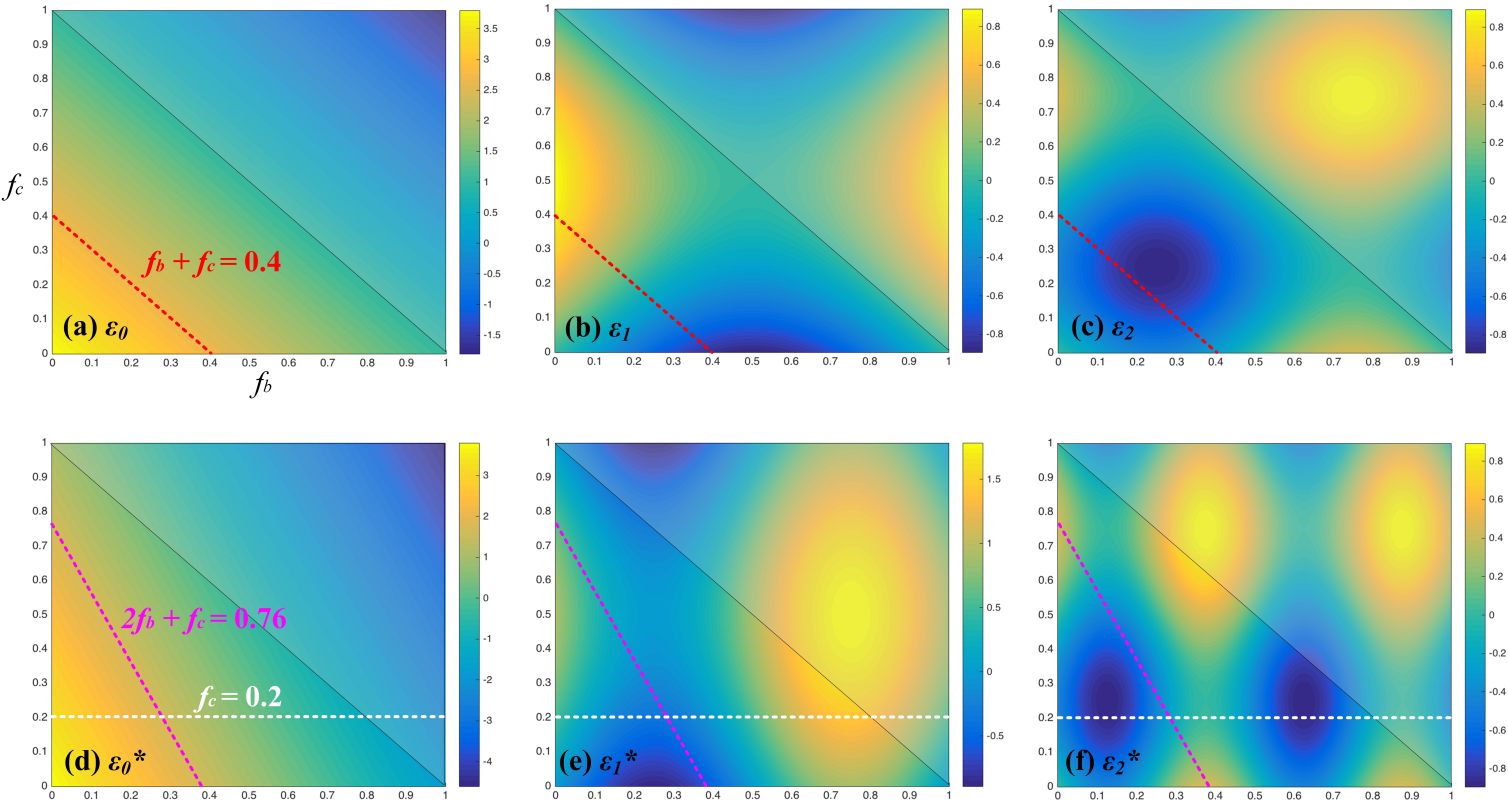
Amplitudes of the grating Fourier harmonics as the function of fill factors $f_{b}$ and $f_{c}$ for the two-part-period grating; (**a**) $\varepsilon_{0}$; (**b**) $\varepsilon_{1}$; and (**c**) $\varepsilon_{2}$; and for the three-part-period grating; (**d**) $\varepsilon_{0}^{*}$; (**e**) $\varepsilon_{0}^{*}$; and (**f**) $\varepsilon_{0}^{*}$. The shadow areas are not significant

Compared with Equation (2), the coefficient in front of $f_{b}$ is doubled due to two free space gaps in each periodic unit. To better understand the modulation property of LSP mode excited in grating, we calculate the amplitude of the first three modes of Fourier harmonics in two-part-period and three-part-period gratings as the function of fill factors $f_{b}$ and $f_{c}$, respectively. Figure 5a–c show the amplitude of $\varepsilon_{0}$, $\varepsilon_{1}$ and $\varepsilon_{2}$ with different fill factors according to Equation (2); the shadow area is not significant with the constraint condition $f_{b}+f_{c}<1$. Since we formulate the $f_{b}+f_{c}=0.4$, $\varepsilon_{0}$ remains unchanged and fixes at about 2.895 (red dotted line in Figure 5a), while $\varepsilon_{1}$ and $\varepsilon_{2}$ obviously vary. This means that the zeroth mode does not contribute to the modulation of absorbing intensity, and, on the contrary, the first and second orders are responsible for the modulation of absorption. Figure 5d–f show the amplitude of $\varepsilon_{0}^{*}$, $\varepsilon_{1}^{*}$, and $\varepsilon_{2}^{*}$, and with different fill factors according to Equation (3). We can find that the slope of $\varepsilon_{0}^{*}$ is enlarged since the function of the zeroth mode is linear; meanwhile, both the variation periods of $\varepsilon_{1}^{*}$ and $\varepsilon_{2}^{*}$ narrow down since the function of them are sinusoidal and the fill factors are phase parameters. In addition, the modulating amplitudes of Fourier harmonics in three-part-period grating, especially the first order, are much better than those in two-part-period grating, which indicates that, by properly varying the fill factors, a wide absorption band over the mid-infrared could probably be realized in this structure.

**Figure 6:**
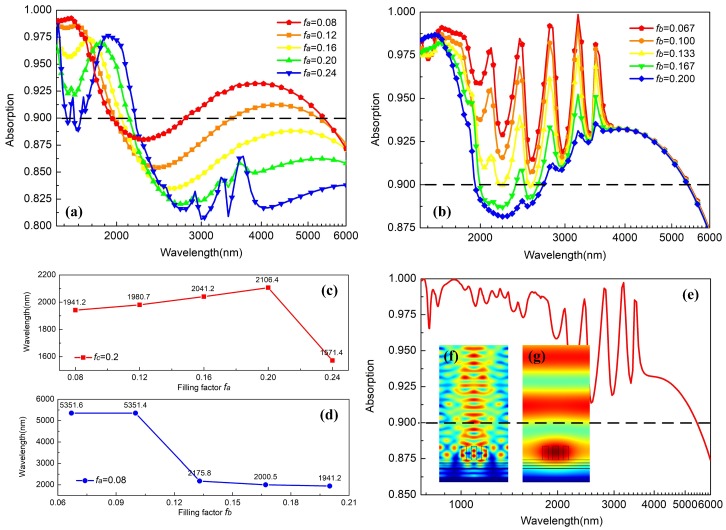
Absorption spectrum of the three-part-period grating with SFSS over the region from 1500 nm to 6000 nm, on the condition of (**a**) fixing $f_{c}=0.2$ and varying $f_{a}$ from 0.08 to 0.24; (**b**) fixing $f_{a}$ = 0.08 and varying $f_{b}$ from 0.200 to 0.067; (**c**,**d**) are the relationship between filling factors and the upper limit of absorption band corresponding to (**a**,**b**), respectively; (**e**) ultra-broadband absorption in our optimized structure from 750.0 nm to 5351.6 nm; electric field distributions at the wavelength of (**f**) 758.4 nm and (**g**) 5003.2 nm.

The calculated absorption performances of the three-part-period grating with three-layer SFSS are depicted in Figure 6. In Figure 6a, fix the value of $f_{c}$ at 0.2 and tune the value of $f_{a}$ from 0.08 to 0.24 (the corresponding value of $f_{b}$ decreases from 0.28 to 0.22); it is obtained that the absorption for incident light in this system reduces as a whole. Note that the relationship of all filling factors here can be expressed as $3f_{a}+2f_{b}+f_{c}=1$; the function curve in this case is plotted as the white dotted line in Figure 5d, thus the modulation of absorption in Figure 6a is highly dependent on the zeroth Fourier harmonic $\varepsilon_{0}^{*}$ and second Fourier harmonic $\varepsilon_{2}^{*}$. In addition, according to the simulation results, we also plot the variation of upper limit of the absorption band as shown in Figure 6c, which slowly grows from 1941.2 nm to 2106.4 nm and then quickly reduces back to 1571.4 nm. Furthermore, as shown in Figure 6b, the absorption can be enhanced a lot if we fix the value of $f_{a}$ at 0.08 and meanwhile tune the value of $f_{b}$ from 0.200 to 0.067. In addition, it is obtained from Figure 6d that the upper limit of AB in our proposed structure can be broadened to 5351.6 nm when $f_{b}=0.067$, which means an effective absorption for incident light wave in the mid-infrared. Furthermore, the corresponding function curve between $f_{b}$ and $f_{c}$ is reported as the pink dotted line ($2f_{b}+f_{c}=0.76$) in Figure 5d. The amplitude of $\varepsilon_{0}^{*}$ remains constant while both the amplitude of $\varepsilon_{1}^{*}$ and $\varepsilon_{2}^{*}$ decrease as $f_{b}$ decreases. This means that, with appropriate design for the three-part grating, the leaky guided modes can be excited solely through the first and second evanescent diffracted order of the grating. Finally, the ultra wide band of absorption in our optimized structure from 750.0 nm to 5351.6 nm is plotted as shown in Figure 6e; in particular, in the near infrared region up to 2158.8 nm, the absorption for light can maintain a level of more than 95%, which exhibits a much better absorbing capacity than other original grating devices. Figure 6f,g show the electric field distribution at the wavelength of 758.4 nm and 5003.2 nm, respectively. As with the distribution in two-part-period grating, the system responds with a powerful LSP resonance over the whole band and PSP resonances in multiple SiO${}_{2}$/Fe layers only contribute to the absorption in the near infrared.

## 4. Conclusions

In summary, we propose and numerically investigate a novel ultra-broadband absorber that is composed of a three-part-period grating coupled multiple Fe/SiO${}_{2}$ layers. Considering the well impedance matching of the Fe stripe to free space, the PSP mode can be enhanced a lot by replacing Au with Fe stripe as well as adding the number of Fe stripes, while the influences of thickness of Fe and upper SiO${}_{2}$ layer on resonance are also analyzed. On the other hand, it is verified that the LSP mode in grating is closely connected with the Fourier harmonics, thus novel two-part-period and three-part-period gratings supported by SFSS are designed and the filling factors are optimized to enhance the LSP resonance mode. In the end, an ultra-broadband absorption from 750.0 nm to 5351.6 nm is realized in our proposed absorber. Thus, our proposed structure possesses great merits in ultra broadband, low costs and fabrication simplicity, which has a variety of potential applications in terms of thermal emitters, optical protective device and solar energy harvesting.
